# The relationship between physical activity and mental health of middle school students: the chain mediating role of negative emotions and self-efficacy

**DOI:** 10.3389/fpsyg.2024.1415448

**Published:** 2024-09-23

**Authors:** Haoming Yan, Ping Huang, Rui Chen, Yicheng Wang

**Affiliations:** ^1^Physical Education Academy (Gymnastics Academy), Chengdu Sport University, Chengdu, China; ^2^Chengdu Railway Middle School Liren Branch, Chengdu, China; ^3^Institute of Sports Training, Chengdu Sport University, Chengdu, China; ^4^Dagang Shanggulin Primary School, Tianjin, China

**Keywords:** physical activity, negative emotion, self-efficacy, middle school student, chain mediation effect

## Abstract

**Objective:**

To explore the relationship between mental health and physical activity (PA) in middle school students, and examining the roles of negative emotions and self-efficacy in the relationship.

**Methods:**

Data from 1,134 Chinese middle school students (50.2% females, 49.8% males; *M*_age_ = 15.18, SD_age_ = 2.00) were collected using the Physical Activity Rating Scale (PARS-3), Positive and Negative Affect Scale (PANAS), General Self-Efficacy Scale (GSES), and Middle School Student Mental Health Scale (MSSMHS).

**Results:**

(1) There is a significant positive correlation between PA and mental health (*r* = 0.16, *p* < 0.01), and the direct path of PA on mental health is significant (*t* = 2.101, *p* < 0.01). (2) PA negatively predicts negative emotions (*r* = −0.12, *p* < 0.01), and is significantly positively correlated with self-efficacy (*r* = 0.24, *p* < 0.01). Negative emotions negatively predict self-efficacy (*r* = −0.23, *p* < 0.01) and mental health (*r* = −0.67, *p* < 0.01). Self-efficacy positively predicts mental health (*r* = 0.30, *p* < 0.01). (3) Negative emotions and self-efficacy play a significant mediating role between PA and mental health. The mediating effect includes three paths: PA → negative emotion → mental health (effect value: 0.130); PA → self-efficacy → mental health (effect size: 0.052); PA → negative emotions → self-efficacy → mental health (effect size: 0.006).

**Conclusion:**

PA among middle school students can indirectly affect mental health through negative emotions and self-efficacy. Middle school students should be encouraged to participate in PA to reduce their negative emotions and increase their self-efficacy, thus improving their mental health.

## 1 Introduction

Mental health issues pose a significant challenge to global public health (World Health Organization, 2020). This is particularly concerning for adolescents, as they experience a high prevalence of mental health problems (Carter et al., 2015). Frequent occurrences of mental health problems among middle school students can often lead to security incidents on campus. Because of their underdeveloped cognitive abilities and restricted problem-solving capabilities, these students might have a higher likelihood of participating in unlawful activities, posing a threat to campus security and societal harmony (Aebi et al., 2014). Meanwhile, mental health problems of secondary school students are also closely related to risky behaviors such as self-harm and suicide (Murdock et al., 2023). Research shows that PA is beneficial to improving mental health (Gmmash et al., 2023). However, many teachers and parents sill ignore PA, the curriculum reform has not achieved the goal of promoting students’ physical and mental health as hoped by the Chinese government (Meng et al., 2021), and the severity of mental health problems among middle school students is escalating (Wu et al., 2022).

PA is good for people’s mental health. PA reduces inflammation through several different processes (inflammation, cytokines, toll-like receptors, adipose tissue, and vagal tone), which can help to improve the health of people with mood disorders, and consequently mental health (Mikkelsen et al., 2017). Empirical studies have demonstrated that PA has a positive effect on the mental health of individuals across all age groups. Surveys conducted during the novel coronavirus pandemic have shown that appropriate PA during isolation can help reduce anxiety and depressive symptoms and enhance well-being in children, thereby improving mental health (Zhao et al., 2023). In addition, research with adolescents has shown that participation in moderate to high-level PA reduces their vulnerability to potential mental health problems and protects against poor mental health, preventing mental health problems (Rodríguez-Romo et al., 2022). Similarly, research with adults suggests that participating in PA can promote mental health by reducing the prevalence, incidence, and duration of mental disorders (Herbert, 2022). Engaging in PA, either solo or within a group, can improve social ties and provide support for older adults while positively influencing their mental health (Seino et al., 2019). Regular PA has been shown to have numerous benefits for middle school students. Nonetheless, research that clearly demonstrates how PA positively influences the mental health of middle school students remains insufficient.

Owing to significant academic stress, middle school students in China frequently experience prolonged periods of negative emotions, including feelings of depression and tension (Ma et al., 2024). In the long run, it could negatively impact mental health, contributing to the onset and exacerbation of mental illnesses and disorders (Iasiello et al., 2019; Jayasankar et al., 2022). Negative emotions can significantly predict mental health problems such as anxiety disorders, depressive symptoms, and problem behaviors (Ramadan et al., 2023). Higher levels of negative emotions can lead to distressing experiences and May interact with negative personality traits such as neuroticism, further damaging an individual’s mental health (Karreman et al., 2013). Individuals who frequently experience negative emotions tend to have lower work efficiency (Xie et al., 2023), reduced life satisfaction (Barlow et al., 2023), difficulties in problem-solving and coping (Yu et al., 2021), and poorer physical and mental health (Lopez and Denny, 2019). Furthermore, one must not overlook the impact of self-efficacy on the mental health of students in middle school. Self-efficacy is closely related to various areas of human practice (Puozzo and Audrin, 2021). Improving self-efficacy in middle school students can aid in alleviating academic stress, minimizing the adverse effects associated with academic pressures and burnout (Chen et al., 2024; Fu et al., 2023), and aid in diminishing symptoms such as depression and anxiety (Tonga et al., 2020). Therefore, self-efficacy plays a crucial role in the mental health of adolescents (Dupéré et al., 2012); when their self-efficacy is low, the risk of encountering mental health issues rises significantly (Yan et al., 2021). Earlier studies have demonstrated that PA positively influences the reduction of negative emotions and enhances self-efficacy (Fathy et al., 2022; Tikac et al., 2021).

**Figure 1:**
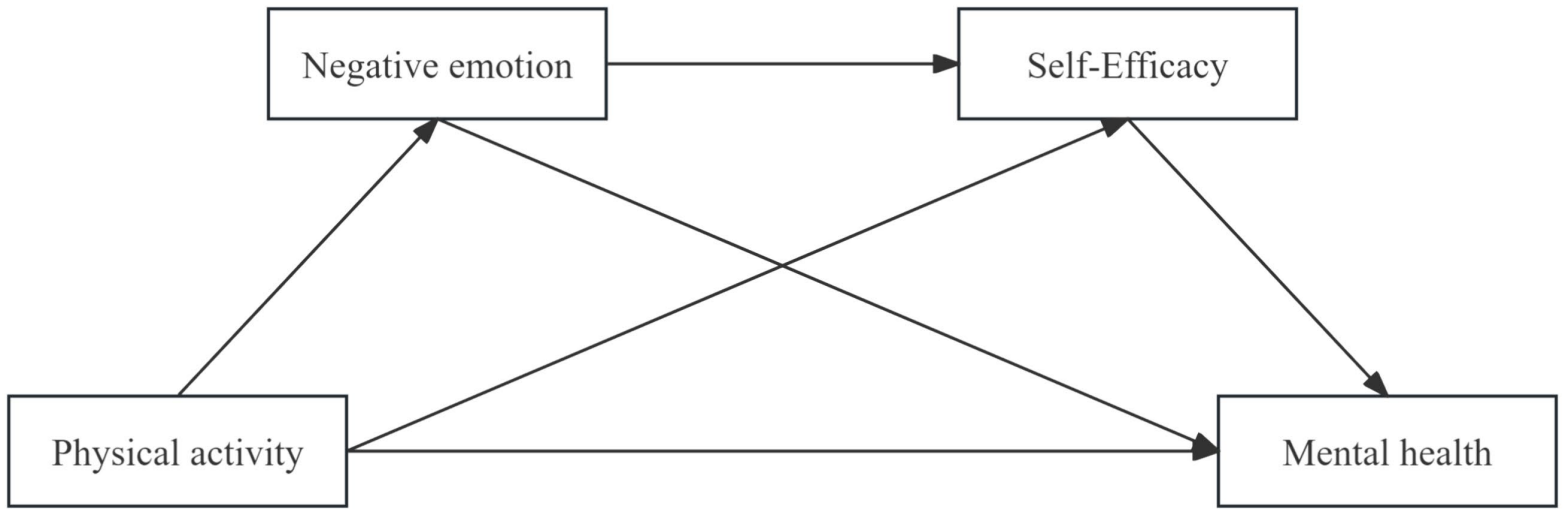
Hypothetical model.

The strength and wisdom of young people are vital for the progress of a nation. Simultaneously, the execution of my nation’s “Outline for Building a Strong Sports Nation” alongside the “National Fitness Plan (2021–2025)” and various other policies has made it crucial to explore effective strategies for improving the mental health of middle school students, which is now a significant subject within the realm of youth education in my country. Consequently, further research is needed to confirm and enhance our understanding of how PA improves mental health. While numerous studies have investigated the relationship between mental health and PA, the majority have concentrated on college students, middle-aged adults, and older adults. However, there is a lack of research on the interconnections and underlying mechanisms between PA and mental health in middle school students. Hence, this study constructed a mediation model of the intrinsic mechanism between PA and mental health (Figure 1), aiming to further explore the relationship between PA and the mental health of middle school students, and provide a theoretical basis for promoting the mental health of middle school students.

In summary, this study proposes the following hypothesis:

*H1*: PA has a positive impact on the mental health of middle school students.

*H2*: Negative emotions play a mediating role between PA and mental health.

*H3*: Self-efficacy acts as a mediator between PA and mental health.

*H4*: Participation in PA influences mental health by mediating the chain effect of negative emotions and self-efficacy.

Figure 1 depicts the model that represents the interplay among these four variables.

## 2 Methods

### 2.1 Participants and procedures

A total of 1,182 students in grades 7 through 12 were sampled from five middle schools in Chengdu, Sichuan Province, through facilitated cluster sampling. In this study, the author team engaged with the school principal and the class teacher in person, clarified the objective of the survey to both the principal and the teacher, and carried out the survey in the classroom following the approval from the principal and class teacher. The questionnaire was first introduced to the students by professionals and informed consent was obtained, and then distributed and collected by professionals with the assistance of class teachers. The data collection period for the questionnaire survey was from February 2024 to March 2024. To ensure data quality, clear identification criteria were established to eliminate invalid questionnaires. The identification criteria for invalid questionnaires included contradictory answers, repetitive filling of relevant characters, and unanswered questions. As a result, 48 invalid questionnaires were excluded, and a total of 1,134 valid questionnaires were collected, yielding an effective rate of 95.9%. Among the valid respondents, 565 were boys (49.8%) and 569 were girls (50.2%). Furthermore, 616 respondents were aged 12–15 (54.3%), while 518 were aged 16–19 (45.7%), the mean age is 15.18 years old and the SD = 2.00. This study obtained ethical approval from Chengdu Sport University (Approval No. CTYLL2024003) and obtained consent from the participating schools and students.

### 2.2 Measuring tools

#### 2.2.1 Physical Activity Rating Scale

The PA status of the research subjects was evaluated using the Chinese version of the physical activity scale revised by Liang (1994). The evaluation was based on three indicators: intensity, frequency, and duration. Each item was quantified using the 5-point Likert scoring method. Intensity and frequency were scored from 1 to 5, indicating weak to strong, while time was scored from 0 to 4, indicating weak to strong. The quantification of PA score was calculated as “intensity × time × frequency.” Higher scores indicate higher levels of PA. In this survey, the Cronbach’s alpha coefficient of the scale was 0.75.

#### 2.2.2 Positive and Negative Affect Scale

The Positive and Negative Emotions Scale, revised by Chen and Zhang (2004), was used to assess the subjects’ emotional state. The scale consists of two dimensions, positive and negative emotions, and the negative emotion dimension has 10 entries (e.g., “upset,” “fearful,” and “hostile”), and this study only used the negative emotion dimension scale to assess the participants’ negative emotions levels. The scale was scored using a five-point Likert scale (1 = almost none, 2 = comparatively little, 3 = moderately much, 4 = comparatively much, 5 = extremely much). Higher scores on the negative affect subscale indicate that participants have more negative affect. In this survey, the Cronbach’s alpha coefficient for the negative affect subscale of the scale was 0.87.

#### 2.2.3 General Self-Efficacy Scale

The General Self-Efficacy Scale translated and revised by Wang et al. (2001) was used, which consists of 10 items (e.g., “I can always solve problems if I try my best,” “I am confident that I can cope effectively with how things come up unexpectedly “). A 4-point Likert scale score was used for quantification (1 = not at all true, 2 = somewhat true, 3 = mostly true, 4 = completely true), with higher scores representing greater perceived self-efficacy on the individual’s part. In this survey, the Cronbach’s alpha coefficient of the scale was 0.90.

#### 2.2.4 Middle School Student Mental Health Scale

The Middle School Student Mental Health Scale, developed by Wang et al. (1997), was used to assess the mental health of Chinese secondary school students based on their cultural characteristics and behavioral tendencies. The scale consists of 60 entries categorized into 10 dimensions, including obsessive-compulsive disorder (e.g., “I have to double-check my homework”), paranoia (e.g., “I always think differently from other people”), hostility (e.g., “I fight with other people”), interpersonal tension and sensitivity (e.g., “I feel that other people are not kind to me”), depression (e.g., “I feel that there is no hope in my future”), anxiety (e.g., “I feel tense or get nervous easily”), academic stress (e.g., “I feel that I have a heavy academic load”), maladjustment (e.g., “I feel uncomfortable with my current school life”), emotional instability (e.g., “I have good and bad moods”), and psychological imbalance (e.g., “I feel sad when my classmates do better than me in exams”). A five-point Likert scale was used (5 = none, 4 = mild, 3 = moderate, 2 = severe, 1 = very severe), with lower scores indicating poorer mental health and more serious mental health problems among participants. In this survey, the Cronbach’s alpha coefficient of the scale was 0.97.

## 3 Data analysis

In order to examine any potential common method deviation, the researchers implemented the Harman single factor method (Podsakoff et al., 2003). The results showed: KMO = 0.97, which produced 13 factors with eigenvalues greater than 1, and a maximum factor variance explained of 30.68%, which is less than the general test of 40%, indicating that there was no significant common method bias in this study. SPSS 27 statistical software was utilized to perform common method deviation testing, description, and correlation analysis. To conduct questionnaire data entry, statistical analysis, and sequential chain mediation effect testing, we utilized the SPSS macro program Process 3.5 plug-in (Model 6) and the Bootstrap method. The PROCESS macro program was employed to analyze the impact of the chain mediation model (Model 6).

## 4 Research results

### 4.1 Descriptive statistics and correlation analysis

The results of the study showed that the average score of PA in the sample was 28.04 ± 22.55, the average score of negative emotion was 19.90 ± 6.91, the average score of self-efficacy was 25.30 ± 6.18, and the average score of mental health was 251.73 ± 37.73 (Table 1).

**Table 1:** Descriptive statistics between variables.

Scale/subscale	*M*	SD
1. Physical activity	28.04	22.55
2. Negative emotion	19.90	6.91
3. Self-efficacy	25.30	6.18
4. Mental health	251.73	37.73
4.1. Obsessive-compulsive disorder	24.07	3.87
4.2. Paranoia	25.68	4.25
4.3. Hostility	26.34	4.46
4.4. Interpersonal tension and sensitivity	25.39	4.43
4.5. Depression	25.30	4.78
4.6. Anxiety	24.97	5.03
4.7. Academic stress	23.93	5.17
4.8. Maladjustment	25.54	4.24
4.9. Emotional instability	24.17	4.53
4.10. Psychological imbalance	26.34	3.66

The Pearson correlation analysis of all research variables in this study is shown in Table 2. PA has a weak but statistically significant negative correlation with negative emotions (*r* = −0.12, *p* < 0.01), indicating that more participation in PA can eliminate negative emotions. PA has a weak positive correlation with self-efficacy (*r* = 0.24, *p* < 0.01), which means that more participation in PA is conducive to the improvement of self-efficacy. PA has a weak positive correlation with mental health (*r* = 0.16, *p* < 0.01), suggesting participating in more PA is beneficial to mental health; negative emotions have a weak negative correlation with self-efficacy (*r* = −0.23, *p* < 0.01), demonstrating that individuals with high levels of negative emotions have low levels of self-efficacy; negative emotions are negatively correlated with mental health (*r* = −0.67, *p* < 0.01), showing that individuals with high levels of negative emotions have more serious mental health problems; self-efficacy is related to mental health is positively correlated (*r* = 0.30, *p* < 0.01), manifesting that individuals with high levels of self-efficacy have a higher level of mental health.

**Table 2:** Results of correlation analysis between variables.

Variable	1	2	3	4
1. Physical activity	1			
2. Negative emotion	−0.12**	1		
3. Self-efficacy	0.24**	−0.23**	1	
4. Mental health	0.16**	−0.67**	0.30**	1

**p < 0.01.

### 4.2 The chain mediating role of mental health, PA, negative emotions and self-efficacy between mental health and PA

In order to conduct additional exploration into the influence of PA on the mental health of middle school pupils, control variables such as gender and age were taken into account. The dependent variable was mental health, while PA was treated as the independent variable. Negative emotions and self-efficacy were used as mediating variables. The mediation effect test was conducted using Hayes’ compiled SPSS macro program, employing Bootstrap method (Hayes, 2017). Model 6 was applied for the model test, with a sample size of 5,000 and a confidence interval of 95%.

Table 3 provides the outcomes of the regression analysis. Mental health is positively and significantly influenced by engaging in PA (β = 0.266, *p* < 0.001), whereas negative emotions are negatively and significantly affected (β = −0.038, *p* < 0.001). When self-efficacy is considered as a dependent variable with PA and negative emotions as predictors, PA (β = 0.059, *p* < 0.001) plays a vital positive role, while negative emotions (β = −0.185, *p* < 0.001) assume a significant negative role. Furthermore, when mental health is predicted using PA, negative emotions, and self-efficacy, both PA (β = 0.078, *p* < 0.001) and self-efficacy (β = 0.887, *p* < 0.001) demonstrate significant positive effects, with negative emotions (β = −0.061, *p* < 0.001) showcasing a strong negative impact.

**Table 3:** Regression analysis of variable relationships in the mediation model.

Regression equation		Overall fit coefficient			Regression coefficient significance	
Outcome variable	Predictor variable	*R*	R2	*F*	β	*t*
Negative emotion	Physical activity	0.123	0.015	17.358	−0.038	−4.167***
Self-efficacy	Physical activity	0.317	0.101	63.282	0.059	7.595***
	Negative emotion				−0.185	−7.303***
Mental health	Physical activity	0.690	0.476	341.709	0.078	2.101**
	Negative emotion				−3.452	−28.452***
	Self-efficacy				0.887	6.360***
Mental health	Physical activity	0.159	0.025	29.459	0.266	5.428***

***p < 0.001.

**Figure 2:**
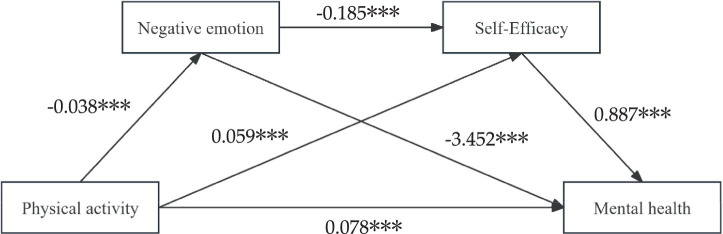
Chain mediation model diagram of negative emotions and self-efficacy. ***p < 0.001.

Table 4 and Figure 2 contain the findings of the analysis conducted on the mediation effect. The value of the total effect is 0.266, and the 95% confidence interval excludes 0, signifying a significant total effect. On the other hand, the direct effect value is 0.078, and the 95% confidence interval does not encompass 0, indicating that PA significantly affects mental health directly. Additionally, the total indirect effect value is 0.188, 95% CI [0.123, 0.255], implying noteworthy mediating effects of negative emotions and self-efficacy between mental health and PA. The mediation effect of the variables negative emotions and mental health consists of three pathways: the indirect effect 1 (effect value 0.130, CI [0.069, 0.193]) is formed by the path of engaging in PA → experiencing negative emotions → achieving mental health, showing a substantial independent impact of negative emotions. The path of PA → enhancing self-efficacy → improving mental health creates an indirect effect 2 (effect value 0.052, CI [0.029, 0.077]), signifying a significant independent mediating effect of self-efficacy. Furthermore, the path of PA → triggering negative emotions → promoting self-efficacy → enhancing mental health demonstrates an indirect effect 3 (effect value 0.006, CI [0.003, 0.011]), revealing a noteworthy mediating effect of negative emotions → self-efficacy. The ratios of the three indirect effects to the total effect were 48.87, 19.55, and 2.26%, respectively. The independent mediating effect of negative emotions had the highest ratio to the total indirect effect.

**Table 4:** Analysis of the mediating effect of mental health and PA.

Direct effect	Effect size	Boot SE	95% confidence interval		Relative mediation effect
			LLCI	ULCI	
Total effect	0.266	0.049	0.170	0.363	100%
Direct effect	0.078	0.038	0.005	0.151	29.30%
Total indirect effect	0.188	0.034	0.123	0.255	70.68%
Indirect effect 1	0.130	0.031	0.069	0.193	48.87%
Indirect effect 2	0.052	0.012	0.029	0.077	19.55%
Indirect effect 3	0.006	0.002	0.003	0.011	2.26%

## 5 Discussion

Students’ mental health is as important as their physical health and is closely related to campus safety, family harmony, and social stability. Improving the mental health of middle school students is now a pressing issue in both education and society as a whole. This study explored the correlation between middle school students’ engagement in PA and their mental health, while also examining the mediating effects of negative emotions and self-efficacy. The study’s findings show that PA can predict mental health both directly and positively. Additionally, PA can indirectly forecast mental health through the independent mediating effects of negative emotions and self-efficacy, as well as through the combined chain mediating effect of both factors. These results carry significant implications for enhancing the mental health of middle school students.

### 5.1 The impact of PA on the mental health of middle school students

Data analysis conducted in this study showed that there is a direct relationship between the extent to which secondary school students participate in PA and their mental health. This finding is consistent with the originally-proposed hypothesis. The results of our study align with those of prior research, demonstrating a direct link between the engagement of high school students in PA and their mental health. Boosting students’ involvement in PA could potentially enhance their mental health (Molcho et al., 2021). Therefore, Hypothesis H1 is confirmed. A study conducted by researchers in China discovered that incorporating music into PA can help foster a positive classroom environment, improve students’ flexibility in coping with psychological challenges, steer students toward healthier psychological inclinations, and ultimately support the mental health of students (Liu, 2024). Enhancing PA levels May also aid in decreasing the general abnormal rate of mental health and easing psychological issues (Dong and Dou, 2023). At the same time, research by Western scholars has also confirmed that PA is beneficial to mental health. PA helps individuals have a more positive emotional response to events and allows individuals to maintain an objective and optimistic attitude toward life (Catalino and Fredrickson, 2011). Furthermore, participation in PA can improve students’ mental health and well-being by increasing their ability to cope with life challenges and setbacks (Malagodi et al., 2024). Participation in sports also promotes positive emotions, self-esteem, and academic performance in adolescents (Rasmussen and Laumann, 2013). In addition, habitual PA are beneficial to brain development and help improve language expression, understanding and cognitive performance (Herting and Chu, 2017). These benefits contribute to the promotion of mental health and overall quality among middle school students. Therefore, middle school students should develop and maintain good PA habits, as PA is an important intervention to alleviate mental health problems when they occur (Klemmer et al., 2023).

### 5.2 The mediating role of negative emotions between PA and mental health

According to this research, it has been discovered that the connection between the mental health of middle school students and PA is mediated by negative emotions. Put simply, engaging in PA helps enhance the mental health of middle school students by diminishing negative emotions. Hypothesis H2 has been confirmed, as supported by prior research. Previous studies have consistently demonstrated that PA play a crucial role in improving mental health by effectively reducing negative emotions (Li J. et al., 2023; Li L. et al., 2023). Research by Chinese scholars shows that sitting for long periods of time will enhance negative emotions such as anxiety and stress among middle school students (Zou et al., 2023), affect the emotional flexibility of middle school students, and then affect mental health (Zhang et al., 2020). Conversely, PA can promote mental health by improving symptoms of depression and stress (Rong et al., 2024). Research by Western scholars has also prove PA can assist individuals in managing their emotional regulation deficiencies, alleviating the enduring negative impact of stressors, and facilitating the elimination of negative emotions (Bernstein and McNally, 2017). This reduction in negative emotions can lead to heightened positive emotional states among students, fostering feelings of cheerfulness, energy, and satisfaction (Reitsema et al., 2023). In summary, PA can enhance students’ attention control, emotional control, life satisfaction, and overall physical and mental health. Moreover, based on physiological theory, engaging in PA can boost activation in the individual’s prefrontal cortex, fortify functional connections across different brain regions, and enhance the flexibility of emotion regulation pathways and nodes, ultimately diminishing the perception of negative emotions and bolstering emotional control (Wang et al., 2024). As a result, it is essential to promote involvement in PA for middle school students during their educational journey in order to reduce negative emotions and improve mental health.

### 5.3 The mediating role of self-efficacy between PA and mental health

The findings indicate that self-efficacy plays a mediating role between PA and mental health, thus confirming Hypothesis 3. This finding is consistent with previous research (Guo and Jiang, 2023; Jia et al., 2022). This study confirms that PA has a positive predictive effect on middle school students’ self-efficacy; self-efficacy has a positive predictive effect on mental health. Studies conducted by Chinese researchers show that adolescents who engage regularly in PA show marked improvements in both social and expressive abilities compared to their peers who are inactive (Li J. et al., 2023; Li L. et al., 2023). Such improvements in social interaction and expression are critical in bolstering self-regulation skills, which, in turn, contribute significantly to higher self-efficacy (Li, 2023). Additionally, participation in PA fosters greater perseverance and consistency of interest among adolescents, which further enhances their self-efficacy (Yu et al., 2024). Studies have shown that individuals with high self-efficacy have stronger psychological flexibility, higher levels of self-admission, self-control, and self-worth (Qin et al., 2023). Interacting with teammates during PA serves to expand adolescents’ cognitive horizons, promote intellectual development, and improve adaptability. These interactions make them more cheerful, energetic, and positive in their outlook, providing a conducive environment for better mental health. Studies conducted by Western researchers support the finding that middle school students’ PA is strongly linked to their life satisfaction as well as their physical and mental health (Zullig and White, 2011). Additionally, self-efficacy has been identified as a significant factor in the connection between PA and mental health (Paxton et al., 2010). At the same time, individuals with high self-efficacy have lower prevalence of depression, higher happiness, and higher levels of mental health (Mak et al., 2011; Paredes et al., 2021).

### 5.4 The chain mediating role of negative emotions and self-efficacy between PA and mental health

The research provided evidence that self-efficacy and negative emotions play a vital role as mediators between PA and mental health. Hypothesis 4 was validated by the results. It was discovered in this study that negative emotions negatively impact self-efficacy, and both factors were identified as crucial determinants of mental health (Hu et al., 2023; Zhang et al., 2024). Research indicates that individuals possessing high levels of self-efficacy are capable of effectively managing and diminishing the expression of their negative emotions (Caro and Popovac, 2021). On the other hand, those who often experience negative emotions are likely to exhibit unrealistic feelings of happiness, set overly emotional goals, show a limited ability to regulate their emotions, and have low self-efficacy. This, consequently, can impact mental health by contributing to mood disorders, behavioral issues, and potentially leading to psychiatric disorders (Clauss et al., 2019). Engaging in PA fosters the development of a positive mindset, mitigates negative emotions, boosts self-esteem, and enhances one’s ability to cope with life’s challenges, thereby promoting a higher sense of self-efficacy and a stronger self-identity (Akdeniz and Kaştan, 2023; De Marco et al., 2023). These improvements also support better interpersonal relationships and social adaptability (Haoran et al., 2023). Consequently, individuals who participate in PA tend to exhibit superior mental health, heightened physical and psychological resilience, and a reduced susceptibility to mental health issues such as anxiety and depression. In conclusion, for middle school students, the connection between PA and mental health should take into account the intermediary impact of feelings of negativity and self-belief.

## 6 Implications

The present research investigates the impact of PA on the mental health of middle school students, focusing on negative emotions and self-efficacy. It offers both theoretical and practical insights for parents and teachers on how to effectively promote the mental health of such students. The study further highlights the significant role of negative emotions in the impact of PA on mental health. Therefore, parents and teachers should actively engage in daily communication with children to understand their emotional experiences. Providing children with adequate opportunities for PA can help alleviate negative emotions and ensure their mental health. Furthermore, the study also emphasizes the importance of self-efficacy in the relationship between PA and mental health. Parents, teachers, and coaches should provide more encouragement, praise, and affirmation to students engaging in PA. This will help students gain more experience in PA and achieve higher levels of sports mastery. Additionally, it will enhance their verbal persuasion abilities and increase their level of self-efficacy (Peura et al., 2021). To begin, parents and teachers should address their negative attitudes toward PA. In China, many parents and teachers believe that PA consume students’ energy and time, hinder their learning progress, and impact their academic performance. This perception needs to be changed, and they should develop a correct understanding of the positive impact of PA on students’ physical and mental health. By adopting a supportive and understanding approach, while minimizing rejection, parents and teachers can encourage students to participate in PA. During sports, it is important for parents, teachers, and coaches to actively listen to the needs of students, while also observing and guiding their emotions. They should also take advantage of the unique characteristics of various sports to foster the comprehensive development of students’ personalities, will qualities, temperaments, and spiritual qualities. In sports such as basketball and football, which rely on teamwork and communication, students May experience negative emotions due to coordination errors. In these situations, it is important for parents, teachers, and coaches to actively guide them. This guidance will help students learn how to regulate their uncomfortable emotions while interacting with their peers and develop and enhance their social skills (Fan, 2023). In the field of future education, it is crucial to prioritize the enhancement of mental health among middle school students. One effective approach to achieve this is by encouraging their active engagement in specific PA that are tailored to meet their needs and promote overall well-being. By taking this approach, we can systematically improve their mental health and contribute to their overall development. By recognizing the significance of mental health and implementing targeted PA programs, we can foster a positive learning environment for middle school students, ensuring their long-term academic success.

## 7 Limitations and strengths

This study has several limitations. Firstly, the sample source is limited to Sichuan Province, China, and has not been expanded to include a wider geographical area. China’s vast territory encompasses significant economic and cultural variations among different regions. Middle school students in these regions May experience varying academic pressures and have different attitudes toward learning and sports. Therefore, future research should take into account factors such as city, family income, and academic pressure. Secondly, as this study is a cross-sectional study, several influencing factors have not yet been explored or considered. Additionally, due to time and resource constraints, the questionnaire used in this study primarily employed the Likert scale to gather responses, without conducting more in-depth interviews. Future research could aim to conduct a more comprehensive investigation of middle school students’ PA and mental conditions, by employing a focused one-to-one model, in order to enhance the depth and breadth of the study. The research subjects of this study are all selected from China, which is important as China’s educational system, educational concepts, and culture are significant contextual factors. Therefore, future research should aim to broaden the scope of the survey and strive to obtain samples from different countries in order to conduct more comprehensive research. In conclusion, while this study acknowledges certain limitations, it effectively establishes that negative emotions and self-efficacy serve as mediating factors between PA and mental health. This relationship enhances our understanding of the mechanisms affecting the mental health of middle school students, offering both theoretical insights and empirical data. Furthermore, the findings of this research offer creative approaches for both educators and parents aiming to enhance mental health results for middle school students, while also laying the groundwork for additional investigation into mental health topics concerning this age group.

## 8 Conclusion

PA has a notable impact on the mental health of middle school students. The relationship between PA and mental health affects the levels of negative emotion and self-efficacy in these students, both directly and indirectly. This implies that engaging in more PA is linked to decreased negative emotions and increased self-efficacy, resulting in overall improvements in mental health. Consequently, focusing on reducing negative emotions and fostering self-efficacy can be beneficial for enhancing the mental health of middle school students.

## Data Availability

The raw data supporting the conclusions of this article will be made available by the authors, without undue reservation.

## References

[ref1] Aebi M. Giger J. Plattner B. Metzke C. W. Steinhausen H. C. (2014). Problem coping skills, psychosocial adversities and mental health problems in children and adolescents as predictors of criminal outcomes in young adulthood. Eur. Child Adolesc. Psychiatry 23, 283–293. doi: 10.1007/s00787-013-0458-y, PMID: 23949100

[ref2] Akdeniz Ş. Kaştan Ö. (2023). Perceived benefit of yoga among adults who have practiced yoga for a long time: a qualitative study. Bio Psycho Soc. Med. 17:19. doi: 10.1186/s13030-023-00276-3 PMC10184339 37189194

[ref3] Barlow M. A. Willroth E. C. Wrosch C. John O. P. Mauss I. B. (2023). When daily emotions spill into life satisfaction: Age differences in emotion globalizing. Psychol. Aging 38, 644–655. doi: 10.1037/pag0000771 37616073 PMC10841306

[ref4] Bernstein E. E. McNally R. J. (2017). Acute aerobic exercise helps overcome emotion regulation deficits. Cognit. Emot. 31, 834–843. doi: 10.1080/02699931.2016.1168284, PMID: 27043051

[ref5] Caro C. Popovac M. (2021). Gaming when things get tough? Examining how emotion regulation and coping self-efficacy influence gaming during difficult life situations. Games Cult. 16, 611–631. doi: 10.1177/1555412020944622

[ref6] Carter T. Guo B. Turner D. Morres I. Khalil E. Brighton E.. (2015). Preferred intensity exercise for adolescents receiving treatment for depression: a pragmatic randomised controlled trial. BMC Psychiatry 15, 247–212. doi: 10.1186/s12888-015-0638-z, PMID: 26467764 PMC4605143

[ref7] Catalino L. I. Fredrickson B. L. (2011). A Tuesday in the life of a flourisher: the role of positive emotional reactivity in optimal mental health. Emotion 11, 938–950. doi: 10.1037/a0024889, PMID: 21859208 PMC3160725

[ref8] Chen Y. Li C. Cao L. Liu S. (2024). The effects of self-efficacy, academic stress, and learning behaviors on self-regulated learning in blended learning among middle school students. Educ. Inf. Technol. doi: 10.1007/s10639-024-12821-w

[ref9] Chen W. F. Zhang J. X. (2004). Structure and validity of the Chinese version of the positive/negative affect scale. Chinese. J. Ment. Health 11:763-765+759. doi: 10.3321/j.issn:1000-6729.2004.11.003

[ref10] Clauss K. Bardeen J. R. Benfer N. Fergus T. A. (2019). The interactive effect of happiness emotion goals and emotion regulation self-efficacy on anxiety and depression. J. Cogn. Psychother. 33, 97–105. doi: 10.1891/0889-8391.33.2.97, PMID: 32746385

[ref11] De Marco J. C. P. Dias D. T. Gonzaga I. Duek V. P. Farias G. O. Martins C. R.. (2023). Effects of teasing in physical education classes, self-efficacy, and physical activity on adolescents’ self-esteem. Psicol. Educ. 29, 185–191. doi: 10.5093/psed2023a13

[ref12] Dong R. B. Dou K. Y. (2023). Changes in physical activity level of adolescents and its relationship with mental health during regular COVID-19 prevention and control. Brain Behav. 13:e3116. doi: 10.1002/brb3.3116, PMID: 37325875 PMC10498090

[ref13] Dupéré V. Leventhal T. Vitaro F. (2012). Neighborhood processes, self-efficacy, and adolescent mental health. J. Health Soc. Behav. 53, 183–198. doi: 10.1177/0022146512442676, PMID: 22660825

[ref1002] Fan X. (2023). Accountability in the evaluation of teacher effectiveness: views of teachers and administrators. Educ. Assess. Eval. Account. 35, 585–611., PMID: 35548074

[ref14] Fathy K. A. Allah Jasim M. A. Jawad H. H. Aljuboory D. S. Al Majidi A. R. J. Rashed Y. A.. (2022). Moderating effect of physical activity between the relation of psychological resilience and negative emotions of university students in Iraq. J. Sport Psychol. 31, 142–152. Available at: https://mail.rpd-online.com/index.php/rpd/article/view/725

[ref15] Fu W. Li Y. Liu Y. Li D. Wang G. Liu Y.. (2023). The influence of different physical activity amounts on learning burnout in adolescents: the mediating effect of self-efficacy. Front. Psychol. 14:1089570. doi: 10.3389/fpsyg.2023.1089570, PMID: 36891208 PMC9986600

[ref16] Gmmash A. Alonazi A. Almaddah M. Alkhateeb A. Sabir O. Alqabbani S. (2023). Influence of an 8-week exercise program on physical, emotional, and mental health in Saudi adolescents: a pilot study. Medicina 59:883. doi: 10.3390/medicina59050883, PMID: 37241115 PMC10223168

[ref17] Guo M. Jiang S. (2023). Structural modeling of EFL/ESL teachers’ physical activity, mental health, psychological well-being, and self-efficacy. BMC Psychol. 11:343. doi: 10.1186/s40359-023-01383-0, PMID: 37853470 PMC10585901

[ref18] Haoran S. Tianci L. Hanwen C. Baole T. Yiran C. Yan J. (2023). The impact of basketball on the social adjustment of Chinese middle school students: the chain mediating role of interpersonal relationships and self-identity. Front. Psychol. 14:1205760. doi: 10.3389/fpsyg.2023.1205760, PMID: 37448718 PMC10338091

[ref19] Hayes A. F. (2017). Introduction to mediation, moderation, and conditional process analysis: A regression-based approach. Guilford Publications.

[ref20] Herbert C. (2022). Enhancing mental health, well-being and active lifestyles of university students by means of physical activity and exercise research programs. Front. Public Health 10:849093. doi: 10.3389/fpubh.2022.849093, PMID: 35548074 PMC9082407

[ref21] Herting M. M. Chu X. (2017). Exercise, cognition, and the adolescent brain. Birth Defects Res. 109, 1672–1679. doi: 10.1002/bdr2.1178, PMID: 29251839 PMC5973814

[ref22] Hu D. Kalokerinos E. K. Tamir M. (2023). Flexibility or instability? Emotion goal dynamics and mental health. Emotion 24, 1078–1091. doi: 10.1037/emo0001318 38127537

[ref23] Iasiello M. A. JosephKeyes C. L. M. Cochrane E. M. (2019). Positive mental health as a predictor of recovery from mental illness. J. Affect. Disord. 251, 227–230. doi: 10.1016/j.jad.2019.03.065, PMID: 30927584 PMC6487880

[ref24] Jayasankar P. Manjunatha N. Rao G. N. Gururaj G. Varghese M. Benegal V.. (2022). Epidemiology of common mental disorders: results from "national mental health survey" of India, 2016. Indian J. Psychiatry 64, 13–19. doi: 10.4103/indianjpsychiatry.indianjpsychiatry_865_21, PMID: 35400745 PMC8992756

[ref25] Jia J. Song L. Li L. (2022). Effects of physical activity on mental health and general self-efficacy of city residents in COVID-19. J. Sport Psychol. 31, 57–66. Available at: http://mail.rpd-online.com/index.php/rpd/article/view/647

[ref26] Karreman A. Van Assen M. A. Bekker M. H. (2013). Intensity of positive and negative emotions: explaining the association between personality and depressive symptoms. Personal. Individ. Differ. 54, 214–220. doi: 10.1016/j.paid.2012.08.040

[ref27] Klemmer B. Kinnafick F. E. Spray C. Chater A. M. (2023). The effectiveness of structured sport and exercise interventions in enhancing the mental health of adolescents with mild to moderate mental health problems: a systematic review. Int. Rev. Sport Exerc. Psychol. 1–24. doi: 10.1080/1750984X.2023.2266823

[ref28] Li B. (2023). The relationship between physical exercise and self-efficacy of college students and the mediating role of emotional self-regulation. J. Sport Psychol. 32, 241–249. doi: 10.2224/sbp.13098

[ref29] Li J. Jiang X. Huang Z. (2023). Exercise intervention and improvement of negative emotions in children: A Meta-analysis. BMC Pediatr. 23:411. doi: 10.1186/s12887-023-04247-z 37608261 PMC10464442

[ref30] Li L. Li Y. Mei Z. (2023). A low degree of physical activity adherence in college students: analyzing the impact of interpersonal skills on exercise adherence in college students. J. Racial Ethn. Health Disparities, 1–10. doi: 10.1007/s40615-023-01747-7 37682424

[ref31] Liang D. Q. (1994). Stress level and its relation with physical activity in higher education. Chin. Ment. Health J. 8, 5–6.

[ref32] Liu Z. (2024). Music teaching curriculum integrates physical exercise to regulate Students' mental health. J. Sport Psychol. 33, 426–435. Available at: https://rpd-online.com/index.php/rpd/article/view/1590

[ref33] Lopez R. B. Denny B. T. (2019). Negative affect mediates the relationship between use of emotion regulation strategies and general health in college-aged students. Personal. Individ. Differ. 151:109529. doi: 10.1016/j.paid.2019.109529

[ref34] Ma A. Tan S. Chen J. Lou H. (2024). Stress events and stress symptoms in Chinese secondary school students: gender and academic year characteristics of the relationship[J]. Front. Public Health 12:1360907. doi: 10.3389/fpubh.2024.1360907, PMID: 38476484 PMC10927803

[ref35] Mak W. W. Ng I. S. Wong C. C. (2011). Resilience: enhancing well-being through the positive cognitive triad. J. Couns. Psychol. 58, 610–617. doi: 10.1037/a0025195, PMID: 21895357

[ref36] Malagodi F. Dommett E. J. Findon J. L. Gardner B. (2024). Physical activity interventions to improve mental health and wellbeing in university students in the UK: a service mapping study. Ment. Health Phys. Act. 26:100563. doi: 10.1016/j.mhpa.2023.100563

[ref37] Meng X. Horrell A. McMillan P. Chai G. (2021). ‘Health First’and curriculum reform in China: the experiences of physical education teachers in one city. Eur. Phys. Educ. Rev. 27, 595–612. doi: 10.1177/1356336X20977886

[ref38] Mikkelsen K. Stojanovska L. Polenakovic M. Bosevski M. Apostolopoulos V. (2017). Exercise and mental health. Maturitas 106, 48–56. doi: 10.1016/j.maturitas.2017.09.003 29150166

[ref39] Molcho M. Gavin A. Goodwin D. (2021). Levels of physical activity and mental health in adolescents in Ireland. Int. J. Environ. Res. Public Health 18:1713. doi: 10.3390/ijerph18041713, PMID: 33578906 PMC7916674

[ref40] Murdock A. R. Rogers M. L. Jackson T. L. Monteiro K. Chambers L. C. (2023). Mental health status of Rhode Island middle school and high school students before versus during the COVID-19 pandemic. J. Sch. Health 94, 489–500. doi: 10.1111/josh.13424 38113526 PMC11088986

[ref41] Paredes M. R. Apaolaza V. Fernandez-Robin C. Hartmann P. Yañez-Martinez D. (2021). The impact of the COVID-19 pandemic on subjective mental well-being: the interplay of perceived threat, future anxiety and resilience. Personal. Individ. Differ. 170:110455. doi: 10.1016/j.paid.2020.110455, PMID: 33071413 PMC7552984

[ref42] Paxton R. J. Motl R. W. Aylward A. Nigg C. R. (2010). Physical activity and quality of life—the complementary influence of self-efficacy for physical activity and mental health difficulties. Int. J. Behav. Med. 17, 255–263. doi: 10.1007/s12529-010-9086-9, PMID: 20449700

[ref43] Peura P. Aro T. Räikkönen E. Viholainen H. Koponen T. Usher E. L.. (2021). Trajectories of change in reading self-efficacy: a longitudinal analysis of self-efficacy and its sources. Contemp. Educ. Psychol. 64:101947. doi: 10.1016/j.cedpsych.2021.101947

[ref44] Podsakoff P. M. MacKenzie S. B. Lee J. Y. Podsakoff N. P. (2003). Common method biases in behavioral research: a critical review of the literature and recommended remedies. J. Appl. Psychol. 88, 879–903. doi: 10.1037/0021-9010.88.5.879 14516251

[ref45] Puozzo I. C. Audrin C. (2021). Improving self-efficacy and creative self-efficacy to foster creativity and learning in schools. Think. Skills Creat. 42:100966. doi: 10.1016/j.tsc.2021.100966

[ref46] Qin L. L. Peng J. Shu M. L. Liao X. Y. Gong H. J. Luo B. A.. (2023). The fully mediating role of psychological resilience between self-efficacy and mental health: evidence from the study of college students during the COVID-19 pandemic. Healthcare 11:420. doi: 10.3390/healthcare11030420 36766995 PMC9914060

[ref47] Ramadan R. Randell A. Lavoie S. Gao C. X. Manrique P. C. Anderson R.. (2023). Empirical evidence for climate concerns, negative emotions and climate-related mental ill-health in young people: a sco** review. Early Interv. Psychiatry 17, 537–563. doi: 10.1111/eip.13374, PMID: 36641809

[ref48] Rasmussen M. Laumann K. (2013). The academic and psychological benefits of exercise in healthy children and adolescents. Eur. J. Psychol. Educ. 28, 945–962. doi: 10.1007/s10212-012-0148-z

[ref49] Reitsema A. M. Jeronimus B. F. van Dijk M. Ceulemans E. van Roekel E. Kuppens P.. (2023). Distinguishing dimensions of emotion dynamics across 12 emotions in adolescents’ daily lives. Emotion 23, 1549–1561. doi: 10.1037/emo0001173, PMID: 36355670

[ref50] Rodríguez-Romo G. Acebes-Sánchez J. García-Merino S. Garrido-Muñoz M. Blanco-García C. Diez-Vega I. (2022). Physical activity and mental health in undergraduate students. Int. J. Environ. Res. Public Health 20:195. doi: 10.3390/ijerph20010195, PMID: 36612516 PMC9819335

[ref51] Rong F. Li X. Jia L. Liu J. Li S. Zhang Z.. (2024). Substitutions of physical activity and sedentary behavior with negative emotions and sex difference among college students. Psychol. Sport Exerc. 72:102605. doi: 10.1016/j.psychsport.2024.102605, PMID: 38346583

[ref52] Seino S. Kitamura A. Tomine Y. Tanaka I. Nishi M. Taniguchi Y. U.. (2019). Exercise arrangement is associated with physical and mental health in older adults. Med. Sci. Sports Exerc. 51, 1146–1153. doi: 10.1249/MSS.0000000000001884, PMID: 30694973 PMC6553972

[ref53] Tikac G. Unal A. Altug F. (2021). Regular exercise improves the levels of self-efficacy, self-esteem and body awareness of young adults. J. Sports Med. Phys. Fitness 62, 157–161. doi: 10.23736/S0022-4707.21.12143-7, PMID: 33555673

[ref54] Tonga J. B. Eilertsen D. E. Solem I. K. L. Arnevik E. A. Korsnes M. S. Ulstein I. D. (2020). Effect of self-efficacy on quality of life in people with mild cognitive impairment and mild dementia: the mediating roles of depression and anxiety. Am. J. Alzheimers Dis. Other Dement. 35:153331751988526. doi: 10.1177/1533317519885264 PMC10623983 31916847

[ref55] Wang C. K. Hu Z. Y. Liu Y. (2001). Research on the reliability and validity of the general self-efficacy scale. Appl. Psychol. 1, 37–40. doi: 10.3969/j.issn.1006-6020.2001.01.007

[ref56] Wang J. S. Li Y. He E. S. (1997). Compilation and standardization of the mental health scale for Chinese middle school students. Soc. Psychol. Sci. 4:6.

[ref57] Wang X. Liu T. Jin X. Zhou C. (2024). Aerobic exercise promotes emotion regulation: a narrative review. Exp. Brain Res. 242, 783–796. doi: 10.1007/s00221-024-06791-1, PMID: 38400992

[ref58] World Health Organization. (2020). APA 7th edition: adolescents health. Available at: https://www.who.int/health-topics/adolescent-health/#tab=tab_1.

[ref59] Wu Z. Wang B. Xiang Z. Zou Z. Liu Z. Long Y.. (2022). Increasing trends in mental health problems among urban Chinese adolescents: results from repeated cross-sectional data in Changsha 2016–2020[J]. Front. Public Health 10:829674. doi: 10.3389/fpubh.2022.829674, PMID: 35284369 PMC8907598

[ref60] Xie W. Ye C. Zhang W. (2023). Negative emotion reduces visual working memory recall variability: a meta-analytical review. Emotion 23, 859–871. doi: 10.1037/emo0001139, PMID: 35951384 PMC9918615

[ref61] Yan Z. Qiu S. Alizadeh A. Liu T. (2021). How challenge stress affects mental health among college students during the COVID-19 pandemic: The moderating role of self-efficacy. Int. J. Ment. Health Promot. 23, 167–175. doi: 10.32604/IJMHP.2021.015937

[ref62] Yu H. Lee L. Popa I. Madera J. M. (2021). Should I leave this industry? The role of stress and negative emotions in response to an industry negative work event. Int. J. Hosp. Manag. 94:102843. doi: 10.1016/j.ijhm.2020.102843

[ref63] Yu H. Zhu T. Tian J. Zhang G. Wang P. Chen J.. (2024). Physical activity and self-efficacy in college students: the mediating role of grit and the moderating role of gender. PeerJ 12:e17422. doi: 10.7717/peerj.17422, PMID: 38803579 PMC11129692

[ref64] Zhang G. Feng W. Zhao L. Zhao X. Li T. (2024). The association between physical activity, self-efficacy, stress self-management and mental health among adolescents. Sci. Rep. 14:5488. doi: 10.1038/s41598-024-56149-4, PMID: 38448518 PMC10917799

[ref65] Zhang Q. Zhou L. Xia J. (2020). Impact of COVID-19 on emotional resilience and learning management of middle school students. Med. Sci. Monit. 26, e924994–e924991. doi: 10.12659/MSM.924994 32869770 PMC7485285

[ref66] Zhao Z. Sarkhani S. Sarkhani M. Imani N. (2023). Effect of a home-based exercise programme on mental health and well-being in children during COVID-19 pandemic. Int. J. Sport Exerc. Psychol. 21, 1006–1023. doi: 10.1080/1612197X.2022.2109186

[ref67] Zou L. Wang T. Herold F. Ludyga S. Liu W. Zhang Y.. (2023). Associations between sedentary behavior and negative emotions in adolescents during home confinement: mediating role of social support and sleep quality. Int. J. Clin. Health Psychol. 23:100337. doi: 10.1016/j.ijchp.2022.100337, PMID: 36199367 PMC9508146

[ref68] Zullig K. J. White R. J. (2011). Physical activity, life satisfaction, and self-rated health of middle school students. Appl. Res. Qual. Life 6, 277–289. doi: 10.1007/s11482-010-9129-z

